# Increase in Suicidal Thinking During COVID-19

**DOI:** 10.1177/2167702621993857

**Published:** 2021-05

**Authors:** Rebecca G. Fortgang, Shirley B. Wang, Alexander J. Millner, Azure Reid-Russell, Anna L. Beukenhorst, Evan M. Kleiman, Kate H. Bentley, Kelly L. Zuromski, Maha Al-Suwaidi, Suzanne A. Bird, Ralph Buonopane, Dylan DeMarco, Adam Haim, Victoria W. Joyce, Erik K. Kastman, Erin Kilbury, Hye-In S. Lee, Patrick Mair, Carol C. Nash, Jukka-Pekka Onnela, Jordan W. Smoller, Matthew K. Nock

**Affiliations:** 1 Department of Psychology, Harvard University; 2 Department of Psychiatry, Massachusetts General Hospital, Boston, Massachusetts; 3 Franciscan Children’s, Brighton, Massachusetts; 4 Department of Biostatistics, Harvard T. H. Chan School of Public Health; 5 Department of Psychology, Rutgers University; 6 National Institute of Mental Health, Bethesda, Maryland

**Keywords:** interpersonal interaction, longitudinal methods, suicide prevention

## Abstract

There is concern that the COVID-19 pandemic may cause increased risk of suicide. In the current study, we tested whether suicidal thinking has increased during the COVID-19 pandemic and whether such thinking was predicted by increased feelings of social isolation. In a sample of 55 individuals recently hospitalized for suicidal thinking or behaviors and participating in a 6-month intensive longitudinal smartphone monitoring study, we examined suicidal thinking and isolation before and after the COVID-19 pandemic was declared a national emergency in the United States. We found that suicidal thinking increased significantly among adults (odds ratio [*OR*] = 4.01, 95% confidence interval [CI] = [3.28, 4.90], *p* < .001) but not adolescents (*OR* = 0.84, 95% CI = [0.69, 1.01], *p* = .07) during the onset of the COVID-19 pandemic. Increased feelings of isolation predicted suicidal thinking during the pandemic phase. Given the importance of social distancing policies, these findings support the need for digital outreach and treatment.

COVID-19 is the largest and most deadly global pandemic the world has experienced in the past century. In addition to its staggering death toll (World Health Organization [WHO], 2020), COVID-19 is expected to have a significant negative impact on mental health (Holmes et al., 2020; Kirzinger et al., 2020). There has been concern that suicide rates may increase (Reger et al., 2020; Thakur & Jain, 2020), and initial findings from Google search trends (Jacobson et al., 2020) and online surveys (Ammerman et al., 2020; Kaparounaki et al., 2020; Killgore et al., 2020) have supported this possibility. Understanding the extent to which suicidal thoughts and behaviors may be increasing during the COVID-19 pandemic is crucial for allocating the necessary resources to mental health interventions to prevent further loss of life.

Previous viral epidemics have been associated with increasing rates of suicidal thoughts and behaviors. Suicide deaths increased during and following the Great Influenza Epidemic of 1918 in the United States and the severe acute respiratory syndrome (SARS) outbreak in Hong Kong among elderly individuals (Cheung et al., 2008; Wasserman, 1992). Furthermore, social distancing to curb the spread of coronavirus may increase social isolation, a well-established risk factor for suicide (Appleby et al., 1999).

The goal of this study was to understand whether suicidal thinking has increased during the COVID-19 pandemic and to test whether this was predicted by increased feelings of social isolation. We collected data on suicidal thinking and social isolation before and during the COVID-19 pandemic using smartphone-based ecological momentary assessment (EMA) and GPS data.

## Method

### Participants

Participants were 55 people with suicidal thinking or a recent suicide attempt who were participating in an ongoing intensive longitudinal study focused on understanding the natural occurrence of suicidal thoughts and behaviors. Participants were recruited in the United States at a large urban hospital emergency department (adult participants, *n* = 24) or adolescent inpatient psychiatry unit (adolescent participants, *n* = 31). Demographic information is reported in Table 1. Adult participants provided written informed consent. Adolescent participants under age 18 provided written assent, and their parents/guardians provided written informed consent.

**Table 1.** Sample Demographic Information (at the Time of Baseline Assessment) Reported Separately for Adult and Adolescent Subsamples Recruited From Separate Sites

Characteristic	Adult sample	Adolescent sample
Age (years)	*M* = 32.04 (*SD* = 11.20)	*M* = 15.25 (*SD* = 1.69)
Gender identity		
Female	14 (58.33)	19 (61.29)
Male	10 (41.66)	11 (35.48)
Nonbinary	0 (0.00)	1 (3.23)
Birth sex		
Female	14 (58.33)	21 (67.74)
Male	10 (41.66)	9 (29.03)
Other or missing	0 (0.00)	1 (3.23)
Race		
White	17 (70.83)	27 (87.10)
Black/African American	3 (12.50)	1 (3.23)
Asian	1 (4.17)	0 (0.00)
Multiracial/other	3 (12.50)	3 (9.68)
Ethnicity		
Hispanic/Latino/Latina	6 (25.00)	2 (6.67)
Sexual identity		
Heterosexual/straight	18 (75.00)	19 (65.52)
Homosexual/gay	2 (8.33)	1 (3.45)
Bisexual	3 (12.50)	7 (24.14)
Other or missing	1 (4.17)	4 (13.79)
Relationship/marital status		
Single, never married	18 (75.00)	—^a^
Living with partner, unmarried	2 (8.33)	—^a^
Married	1 (4.17)	—^a^
Separated or divorced	3 (12.50)	—^a^

Note: Values are *n*s (with percentages in parentheses) unless otherwise indicated. Baseline assessment occurred at the time of study enrollment, which for many participants was months before March 2020.

a Relationship/marital status was not collected for adolescent sample.

Inclusion criteria were fluency in English, presentation at hospital with suicidal thoughts, cognitive capacity to provide informed consent as judged by a clinician on the individual’s treatment team, and possession of a smartphone. Inclusion criteria for the current study were as follows: The participants were enrolled as of March 13, 2020; participants had to have completed at least one EMA survey at least 1 week prior (March 6, 2020); and participants had to have completed at least one survey after March 13. Of the 71 participants who were participating in the study in March 2020, 10 were excluded because they had no survey data on or after March 13, four were excluded because they had no data before March 13, and two were excluded because they completed their initial surveys less than 1 week before March 13 (and thus did not provide an adequate baseline).

### Procedures

Demographic data, including race, ethnicity, gender identity, and birth sex, were assessed through participant self-report at the time of study enrollment. Participants were sent smartphone-based surveys assessing presence and severity of suicidal thinking six times per day for the first 3 months of their participation and then once per day for the following 3 months. Morning and evening surveys were sent at scheduled times, and daytime prompts were sent randomly within predefined intervals. Surveys asked participants to rate their current suicidal urges and intent (0–10), summed to create a suicidal thinking score (range = 0–20) at each assessment. Participants also rated how isolated they felt (0–10).

An objective measure of social isolation was additionally quantified using GPS data gathered passively from a subset of participants’ smartphones (*n* = 25; seven adults and 18 adolescents) using the Beiwe app (Torous et al., 2016). After imputation of missing data, we computed hours spent at home per day per participant. Additional information about assessments and GPS data collection is provided in the Supplemental Material available online. All procedures were approved by the governing hospital/university institutional review boards in accordance with the provisions of the World Medical Association Declaration of Helsinki.

### Data analysis

We used March 13, 2020, the date on which COVID-19 was declared a national emergency in the United States, as the cutoff date separating the prepandemic and pandemic phases. Data included in these analyses were collected from the time period between October 8, 2019, and April 17, 2020. We used ordinal flexible-threshold mixed models (a) to test whether levels of suicidal thinking and isolation changed as a function of pandemic phase and (b) to test the interactive effects of pandemic phase, recruitment site (adult vs. adolescent), and self-reported isolation. Third, we used a generalized additive mixed model (GAMM) to test for nonlinear continuous trend effect of time alongside a categorical effect of pandemic phase divided at March 13. Finally, we used linear mixed-effects models to test whether GPS data showed participants isolating at home more after March 13 and whether time spent at home predicted suicidal thinking.

Mixed (or multilevel) models were used because they allow for correlations and/or unequal variances between within-persons error terms as well as unequal numbers of data points across participants, allowing the use of all participants’ data despite variable degrees of missingness. To control for correlations among observations within individuals, we entered participant as a random effect in all analyses.

All analyses of survey data were run in the R software environment (Version 4.0.0; R Core Team, 2020), with the exception of GPS data, which were analyzed in Python using the Spyder environment. We used the R packages *ordinal* (Version 2019-12-12; Christensen, 2019) for estimation of the ordinal flexible-threshold mixed models, *lme4* (Version 1.1-21; Bates et al., 2015) for estimation of linear mixed models, and *mgcv* (Version 1.8-28; Wood, 2017, 2019) for estimation of the GAMMs.

## Results

**Fig. 1.**
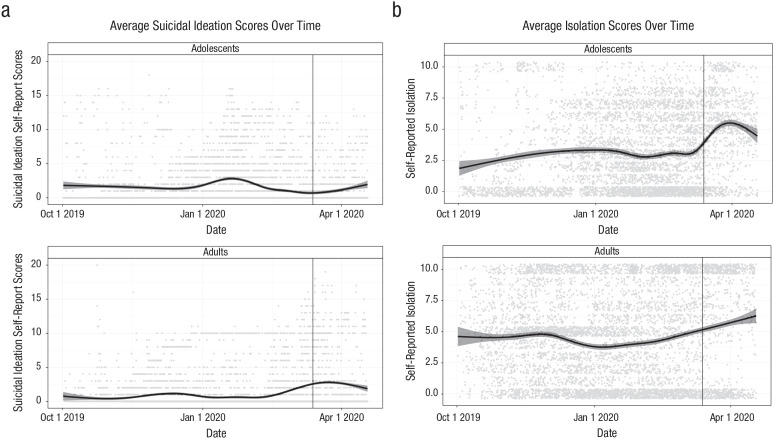
Average self-reported (a) suicidal ideation and (b) isolation scores over time in adolescents (top panels) and adults (bottom panels). The vertical lines represent March 13, 2020, separating the prepandemic phase from the pandemic phase.

Participants provided a total of 12,251 observations. Suicidal thinking increased from the prepandemic phase to pandemic phase (odds ratio [*OR*] = 1.73, 95% confidence interval [CI] = 1.51, 1.99, *p* < .001). A second model including recruitment site (adult vs. adolescent) as a predictor revealed a significant interaction between pandemic phase and site (*OR* = 5.01, 95% CI = [3.79, 6.62], *p* < .001) such that the increase in suicidal thinking was observed among adults (*OR* = 4.01, 95% CI = [3.28, 4.90], *p* < .001)^1^ but not adolescents (*OR* = 0.84, 95% CI = [0.69, 1.01], *p* = .067; Fig. 1a). Visual inspection of the data reveals a brief increase in suicidal thinking among adolescents beginning in January, which then decreases leading up to the pandemic.

Feelings of isolation also significantly increased from the prepandemic phase to the pandemic phase (*OR* = 2.96, 95% CI = [2.57, 3.11], *p* < .001; Fig. 1b), and this effect did not differ by site. There was a significant interaction between pandemic phase and isolation in predicting suicidal thinking in the full sample: Isolation had a stronger association with suicidal thinking during the pandemic phase than it did before the pandemic phase (*OR* = 1.10, 95% CI = [1.05, 1.15], *p* < .001).

**Fig. 2.**
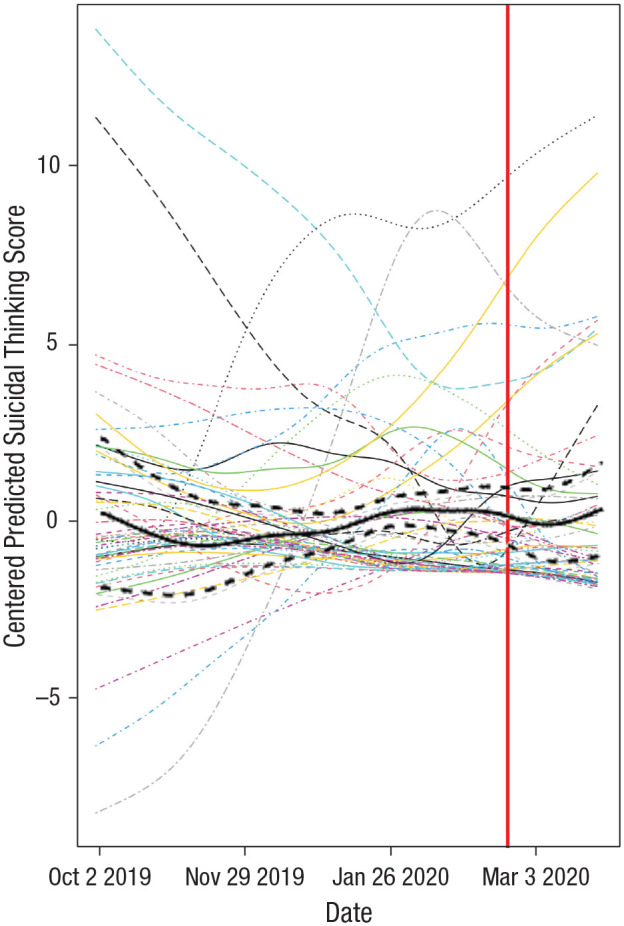
Suicidal thinking trajectories. This plot shows the changes in suicidal thinking scores over time as a smoothed function on average across participants (bold black line) as well as for each individual participant (light dashed and solid lines). The dashed bold black lines indicate the 95% confidence interval. The vertical line represents March 13, 2020, separating the prepandemic phase from the pandemic phase.

Results from the GAMM also showed a significant effect of pandemic phase (coefficient estimate = 0.45, *p* = .003) and a significant nonlinear effect of time (estimated *df* = 7.13, reference *df* = 7.13, *F* = 2.19, *p* = .025). There was substantial variability across individuals in smoothed trajectories of suicidal ideation over time (Fig. 2). When examining average levels of suicidal thinking before and during the pandemic phase, among adults, we found that 54.17% had increases in the pandemic phase, 37.50% had decreases, and 8.33% had no change. Among adolescents, 35.48% had increases, 58.06% had decreases, and 6.45% had no change.

**Fig. 3.**
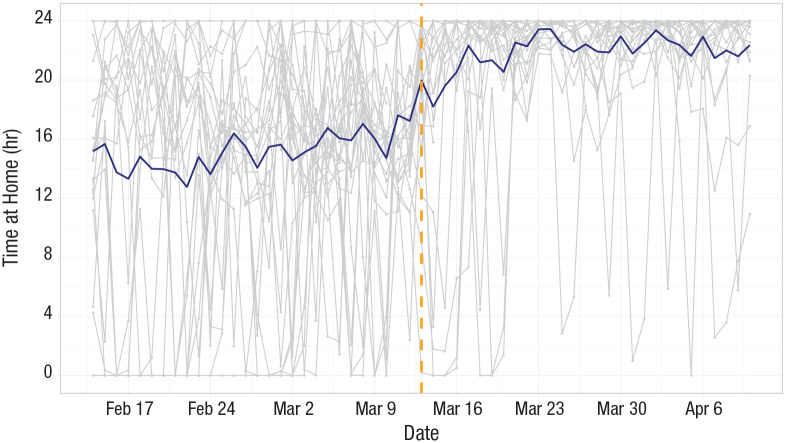
Hours spent at home per day. The blue line represents average across participants each day, and the gray lines represent individual participants. Vertical dashed line is March 13, 2020, the start of the pandemic phase, and plot shows 35 days before and after this date.

GPS data were available from 25 participants and revealed that they spent significantly more hours per day at home during the pandemic phase than the prepandemic phase (unstandardized regression coefficient [*b*] = 7.18, 95% CI = [6.38, 7.97], *p* < .001, Fig. 3). However, time spent at home was not predictive of suicidal thinking (*b* = −0.02, 95% CI = [−0.04, 0.01], *p* = .143). Full results of all models estimated are reported in the Supplemental Material.

## Discussion

This study revealed a significant longitudinal increase in suicidal thinking among adults during the COVID-19 pandemic, which was predicted by a corresponding increase in self-report feelings of social isolation but not GPS measures of time spent at home. Adolescents reported significant increases in isolation but not suicidal thinking during the pandemic. These findings support concerns that one important mental health consequence of the response to COVID-19 may be increased risk of suicidal thoughts for some individuals.

Two findings warrant further investigation. First, the lack of an average increase in suicidal thinking among adolescents was surprising and should be studied further. It may be that many adolescents did not experience a pandemic-associated increase because of greater social connectedness via social media; less stress related to school/work, financial strain, or housing; less attention to news reports about COVID-19; or symptom improvements related to the treatment they received on the inpatient unit. Adolescents did show an observable increase in suicidal thinking in late January, potentially because of the start of school, that subsided before the pandemic phase.

Second, although suicidal thinking increased on average, the level of suicidal thinking remained relatively low both before and during the pandemic phase. The results also reflected substantial variability both between and within participants. The variability in responses to the pandemic suggests a need for follow-up investigations of factors that may moderate variation in response, such as personal experience with COVID-19, employment status, presence of others in the home, access to mental health services, experience of racism, substance use, psychological traits, and distinct phenotypes of suicidal thought trajectory at baseline (Kleiman et al., 2018).

These findings should be interpreted in the context of several limitations. First, the current data do not speak to potential increases in suicidal behaviors. We did not have sufficient power in the current sample to test for changes in suicide attempts. Data from the Massachusetts Department of Health Registry of Vital Records and Statistics demonstrate that deaths by suicide did not deviate from expected rates in March through May of 2020, when the stay-at-home advisory was in effect (Faust et al., 2020). Although it is reassuring that suicide deaths did not increase during the stay-at-home advisory in Massachusetts, we believe that increased focus on suicide risk assessment and prevention is warranted given the current findings.

In addition, these data are from a relatively small monitoring study from one geographic region in patients with recent history of suicidal thoughts or attempt. It is unclear whether the observed increases have occurred in other regions or whether they reflect increases above and beyond typical seasonal variation. Moreover, these data do not speak to potential increased suicidal thinking among the elderly, given that no participants were over 60, or whether the pandemic may be associated with increases in the onset of suicidal thoughts among those who have never had them. Examination of different age groups was confounded with recruitment site, and although no differences in severity of suicidal thinking were observed between sites, there may be differences between sites that complicate analyses comparing adults with adolescents. Furthermore, we focused on only the early stages of the pandemic’s effect on the daily lives of individuals in the United States. Just as there have been waves of infection and other outcomes associated with COVID-19, there may have been—and continue to be—waves of psychological responses to the pandemic. Future work may explore changes in suicidal thinking during later stages of the pandemic. Finally, there was substantial missingness in EMA survey data, and GPS data were available for only a subset of participants.

Despite these limitations, the current findings shed light on changes in suicidal thinking during COVID-19. Understanding risk factors for suicide during this time may facilitate the development of targeted interventions to prevent further loss of life. Given the health requirements for social distancing because of this pandemic, these results suggest an increased need for digital outreach and virtual psychiatric care with a focus on suicide risk assessment and prevention.

## Supplemental Material

sj-pdf-1-cpx-10.1177_2167702621993857 – Supplemental material for Increase in Suicidal Thinking During COVID-19 Click here for additional data file. Supplemental material, sj-pdf-1-cpx-10.1177_2167702621993857 for Increase in Suicidal Thinking During COVID-19 by Rebecca G. Fortgang, Shirley B. Wang, Alexander J. Millner, Azure Reid-Russell, Anna L. Beukenhorst, Evan M. Kleiman, Kate H. Bentley, Kelly L. Zuromski, Maha Al-Suwaidi, Suzanne A. Bird, Ralph Buonopane, Dylan DeMarco, Adam Haim, Victoria W. Joyce, Erik K. Kastman, Erin Kilbury, Hye-In S. Lee, Patrick Mair, Carol C. Nash, Jukka-Pekka Onnela, Jordan W. Smoller and Matthew K. Nock in Clinical Psychological Science

## Transparency

*Action Editor:* Aidan G. C. Wright

*Editor:* Kenneth J. Sher

*Author Contributions*

M. K. Nock, E. M. Kleiman, S. A. Bird, R. Buonopane, J.-P. Onnela, and J. W. Smoller obtained funding and designed the larger study. M. K. Nock, R. G. Fortgang, A. J. Millner, E. M. Kleiman, K. H. Bentley, K. L. Zuromski, S. A. Bird, R. Buonopane, C. C. Nash, and J. W. Smoller structured and oversaw data collection. M. K. Nock, E. Kilbury, M. Al-Suwaidi, H. S. Lee, V. W. Joyce, D. DeMarco, and E. K. Kastman assisted with data collection and management. R. G. Fortgang, S. B. Wang, A. J. Millner, A. Reid-Russell, A. L. Beukenhorst, P. Mair, and M. K. Nock designed and conducted the analyses. R. G. Fortgang drafted the initial version of the manuscript. All of the authors revised and approved the final manuscript for submission.

## Notes

1. This finding remains significant when restricting included data to an equal number of included days before and after March 13.
