# Supplementary material for: Increase in Suicidal Thinking During COVID-19
Source: Clin Psychol Sci. 2021 May;9(3):482–8. doi: 10.1177/2167702621993857 (PMC7967020; doi:10.1177/2167702621993857)
Supplement: sj-pdf-1-cpx-10.1177_2167702621993857 – Supplemental material for Increase in Suicidal Thinking During COVID-19 [file sj-pdf-1-cpx-10.1177_2167702621993857.pdf]

Supplemental Online Material

Supplemental Methods

Assessment

Prompts were delivered and responses collected via LifeData software. Participants were compensated with $1 per survey. To assess suicidal thinking, we used two items. The first, "Right now, how strong is your urge to kill yourself?", was rated from 0 ("not at all") to 10 ("very strong"). The second, "Right now, how strong is your intention to kill yourself today?" was rated from 0 ("I am definitely not going to kill myself today") to 10 ("I am definitely going to kill myself today"). These two items were summed to create a suicidal thinking score ranging from 0-20. In instances of ratings of 8 or above on suicidal intent, a clinical psychologist on our team followed up with the participant to conduct a risk assessment by phone. To rate feelings of isolation, we used the following item: "Right now, how much do you feel isolated from others?" rated from 0 ("not at all") to 10 ("very much").

After three days of no survey data provided by a participants, study staff attempted to contact the participant up to five times over the course of 26 days prior to considering the participant lost to follow up. After that point, the participant was still permitted to reengage without prompting until their study completion date (six months after they began the study).

Location

Location data were collected using the Beiwe smartphone application.[1] To conserve smartphone battery life, location data were-collected for 90 seconds every 15 minutes. Latitude-longitude coordinate pairs were projected on a 2D plane, then converted into a temporal sequence of flights (periods of linear movement) and pauses (stationary periods), and missing data were imputed using the method described previously.[2] From the imputed data, we calculated

daily time spent at home (in hours) per day per participant. Sufficient location data were available for 25 participants. Of the remaining 29 participants, two participants declined to participate in this component of the study at the time of enrolling. Of participants who agreed to this portion of the study, no data were available for five, the app stopped gathering data partway through study participation and before the pandemic phase for 14, and data quality were too low (too few location records were recorded) for imputation of missing data for nine. This level of missingness resulted from the fact that the application was not used for surveys and was running purely in the background; in this situation, the operating system will typically shut it down in order to conserve phone battery, CPU usage, and memory.

Supplemental Results

Full results and model comparisons of all ordinal flexible-threshold mixed models testing the associations between pandemic phase and site with self-reported suicidal thinking are reported in **Table S1**. In Model 1, pandemic phase was entered as a sole predictor. In Model 2, recruitment site (adolescents vs. adults) was entered as an additional predictor. In Model 3, the interaction between recruitment site and pandemic phase was entered as an additional predictor. Site did not significantly predict suicidal thinking, whereas the interaction of pandemic phase did, and likelihood ration tests revealed that Model 3 significantly improved model fit.

Full results and model comparisons of ordinal flexible-threshold mixed models testing the association between pandemic phase and social isolation are reported in **Table S1**. These models tested the effect of pandemic phase alone on social isolation (Model 4), the addition of recruitment site (adolescents vs. adults (Model 5), and the addition of their interaction (Model 6). Likelihood ratio tests demonstrate that adding parameters for site or the interaction of site and pandemic phase did not significantly improve model fit.

Full results and model comparisons of all ordinal flexible-threshold mixed models testing the association between social isolation and self-reported suicidal thinking are reported in **Table S2**. These models tested the effect of social isolation alone on suicidal thinking (Model 7), the addition of pandemic phase (Model 8), and the addition of their interaction term (Model 9). Likelihood ratio tests indicated that Model 6 was the superior fitting model.

There was substantial variability in how many data points were contributed among participants (average number of surveys per included participant = 346, range = 17-636, overall percentage of surveys completed 43.60%). Each participant was sent six surveys per day for the three months after presentation at the hospital and then one survey per day for the following three months. All participants had missing data and we made the choice *a priori* to include all possible participant data regardless of whether they had completed their 6 months of participation. Additionally, due to a glitch in the survey-delivery app 1 participant received twice as many survey prompts as intended during a portion of study participation, resulting in a contribution of more data points than any other participant. We ran all analyses with and without this participant included, and the pattern of significant results in all analyses were the same with one exception: the non-linear effect of time and categorical effect of pandemic phase was no longer significant using a GAMM.

Results of linear mixed models testing for effect of pandemic phase on hours spent at home and effect of hours spent at home on suicidal thinking are reported in **Table S3**.

Additionally, our pattern of primary results was unchanged when using only an item assessing suicide urge rather than the sum of suicide urge with intent.

Supplemental Tables

**Predicting Self-Reported Suicidal Thinking**			**Predicting Self-Reported Isolation**		
**Model 1 – Pandemic Phase Only**			**Model 4 – Pandemic Phase Only**		
Fixed effects	*OR (95% CI)*	*p*	*Fixed effects*	*OR (95% CI)*	*p*
Pandemic phase	1.73 (1.51-1.99)	<0.001	Pandemic phase	2.78 (2.48-3.11)	<0.001
Random effects			*Random effects*		
σ^2	3.29		σ^2	3.29	
$\tau_{00\ subn}$	5.39		$\tau_{00\ subn}$	3.24	
ICC	0.62		ICC	0.50	
AIC	24841		AIC	46625	
Log Likelihood	-12398		Log Likelihood	-23301	
**Model 2 – Pandemic Phase and Site**			**Model 5 – Pandemic Phase and Site**		
Fixed effects	*OR (95% CI)*	*p*	*Fixed effects*	*OR (95% CI)*	*p*
Pandemic phase	1.75 (1.52-2.01)	<0.001	Pandemic phase	2.78 (2.48-3.11)	<0.001
Site [Adults]	0.66 (0.18-2.42)	0.531	Site [Adults]	1.30 (0.52-3.25)	0.572
Random effects			*Random effects*		
σ^2	3.29		σ^2	3.29	
$\tau_{00\ subn}$	5.39		$\tau_{00\ subn}$	3.21	
ICC	0.62		ICC	0.49	
AIC	23011		AIC	46627	
Log Likelihood	-11493		Log Likelihood	-23300	
**Model 3 – Pandemic Phase, Site, and Interaction**			**Model 6 – Pandemic Phase, Site, and Interaction**		
Fixed effects	*OR (95% CI)*	*p*	*Fixed effects*	*OR (95% CI)*	*p*
Pandemic phase	0.83 (0.69-1.01)	0.064	Pandemic phase	2.96 (2.57-3.41)	<0.001
Site [Adults]	0.48 (0.14-1.60)	0.231	Site [Adults]	1.34 (0.48-3.73)	0.577
Pandemic Phase * Site	5.01 (3.79-6.6207)	<0.001	Pandemic Phase * Site	0.85 (0.67-1.07)	0.157
Random effects			*Random effects*		
σ^2	3.29		σ^2	3.29	
$\tau_{00\ subn}$	5.38		$\tau_{00\ subn}$	3.20	
ICC	0.62		ICC	0.49	
AIC	22884		AIC	46627	
Log Likelihood	-12398		Log Likelihood	-23299	
**Model Comparison**			**Model Comparison**		
	Likelihood Ratio (df)	*p*		*Likelihood Ratio (df)*	*p*
Model 1/Model 2	-1811.70 (9)	1.00	*Model 4/Model 5*	0.29 (1)	0.593
Model 1/Model 3	-1941.00 (8)	1.00	*Model 4/Model 6*	2.285 (2)	0.319
Model 2/Model 3	129.29 (1)	<0.001	*Model 5/Model 6*	2.00 (1)	0.157

Table S1. Full results of series of nested flexible threshold ordinal mixed models testing effects of pandemic phase and site on self-reported suicidal thinking and social isolation.

Model 7 – Isolation Only		
Fixed effects	*OR (95% CI)*	*p*
Isolation	1.34 (1.31-1.36)	<0.001
Random effects		
σ^2	3.29	
$\tau_{00\ subn}$	4.67	
ICC	0.59	
AIC	23879	
Log Likelihood	-11918	
**Model 8 – Isolation and Pandemic Phase**		
Fixed effects	*OR (95% CI)*	*p*
Isolation	1.33 (1.31-136)	<0.001
Pandemic phase	1.39 (1.20-1.60)	<0.001
Random effects		
σ^2	3.29	
$\tau_{00\ subn}$	4.74	
ICC	0.59	
AIC	23861	
Log Likelihood	--11908	
**Model 9 – Isolation, Pandemic Phase, and Interaction**		
Fixed effects	*OR (95% CI)*	*p*
Isolation	1.31 (1.28-1.34)	<0.001
Pandemic phase	0.74 (0.53-1.05)	0.090
Isolation * Pandemic phase	1.10 (1.01-1.15)	<0.001
Random effects		
σ^2	3.29	
$\tau_{00\ subn}$	4.69	
ICC	0.59	
AIC	23847	
Log Likelihood	-11899	
**Model Comparison**		
	Likelihood Ratio (df)	*p*
Model 7/Model 8	19.797 (1)	<0.001
Model 7/Model 9	36.398 (2)	<0.001
Model 8/Model 9	16.601 (1)	<0.001

Table S2. **Full results of series of nested flexible threshold ordinal mixed models testing effects of social isolation and pandemic phase on self-reported suicidal thinking.**

Model 10 – Pandemic Phase Predicting Home Time		
Fixed effects	*B (95% CI)*	*p*
Pandemic Phase	7.18 (6.38-7.97)	<0.001
Random effects		
σ^2	33.58	
$\tau_{00\ subn}$	7.36	
ICC	0.18	
**Model 11 – Home Time Predicting Suicidal Thinking**		
Fixed effects	*B (95% CI)*	*p*
Home Time	-0.02 (-0.04-0.01)	0.143
Random effects		
σ^2	4.20	
$\tau_{00\ subn}$	5.03	
ICC	0.54	

Table S3. **Full results of linear mixed models testing effect of pandemic phase (starting March 13, 2020) on home time (hours spent at home per day), and testing effect of home time on suicidal thinking scores.**

Supplemental References

1. Torous, J., Kiang, M. V., Lorme, J. & Onnela, J.-P. New Tools for New Research in Psychiatry: A Scalable and Customizable Platform to Empower Data Driven Smartphone Research. *JMIR Mental Health* **3**, e16 (2016).
2. Barnett, I. & Onnela, J.-P. Inferring mobility measures from GPS traces with missing data. *Biostatistics* **21**, e98–e112 (2020).
